# Lack of usefulness of computed tomography for surveillance in patients with aggressive non-Hodgkin lymphoma

**DOI:** 10.1371/journal.pone.0192656

**Published:** 2018-02-14

**Authors:** Ka-Won Kang, Se Ryeon Lee, Dae Sik Kim, Eun Sang Yu, Hwa Jung Sung, Seok Jin Kim, Chul Won Choi, Yong Park, Byung Soo Kim

**Affiliations:** 1 Division of Hematology-Oncology, Department of Internal Medicine, Korea University College of Medicine, Seoul, South Korea; 2 Division of Hematology-Oncology, Department of Internal Medicine, Sungkyunkwan University School of Medicine, Seoul, South Korea; Universita degli Studi di Roma La Sapienza, ITALY

## Abstract

Surveillance computed tomography (CT) is usual practice for patients with aggressive non-Hodgkin lymphoma (aNHL) in complete remission (CR). However, evidence to support this strategy is lacking. We retrospectively analyzed our institutional lymphoma registry, including patients with lymphoma consecutively enrolled from June 1995 to October 2016. Of 1,385 patients with aNHL, 664 achieved CR and were followed up with or without surveillance CT. Surveillance CT was performed for 609 patients every 3 or 6 months for the first 2 years, then every 6 or 12 months thereafter. Relapse was detected in 171 patients, of whom 152 underwent surveillance CT during follow-up. Of these 152 patients, asymptomatic relapse was detected in 67 (44%) using surveillance CT, and symptomatic relapse outside the surveillance interval was detected in the remaining 85 (56%). Detection of asymptomatic relapse using surveillance CT did not improve the overall or post-relapse survival in patients with relapsed aNHL. Surveillance CT interval (3 or 6 months) did not affect survival. No subgroups were identified that favored the use of surveillance CT to detect relapse. The results of this study suggest that routine surveillance CT in patients with aNHL to detect asymptomatic relapse might have a limited role in improving survival. CT is recommended when a relapse is clinically suspected.

## Introduction

The use of surveillance computed tomography (CT) in patients with aggressive non-Hodgkin lymphoma (aNHL) who have achieved complete remission (CR) is generally recommended in the guidelines of various medical professional societies [1–4]. The rationale for this recommendation is based on the belief that surveillance CT might contribute to the early detection of asymptomatic relapse, which might translate to a favorable clinical outcome in patients with relapsed aNHL. However, this expectation is not strongly supported by a high level of evidence. Rather, conflicting results about the benefits of surveillance CT in patients with aNHL in CR have been reported [5–7].

The use of surveillance CT poses potential risks for the patient. The most concerning risk is the development of a secondary malignancy owing to radiation exposure. Several studies have indicated that the low-dose radiation incurred from CT is significantly associated with the development of cancer and only 2–3 scans have been shown to result in an increased risk of cancer [8–10]. This risk is higher in younger patients than in older patients and higher in women than in men [11].

Surveillance CT may lower patient quality of life. A study reported that recurrent cancer was one of the major concerns in patients with lymphoma, and increased anxiety was associated with surveillance CT [12]. There are also potential risks related to the use of contrast media, such as acute kidney injury, allergic reaction, and anaphylaxis, among others. These events might lead to critical situations in some cases, requiring considerable medical intervention. Finally, the use of surveillance CT during CR follow-up significantly increases the overall cost of health care [13].

Considering the medical, psychological, and socioeconomic risks of surveillance CT, the benefit of this strategy remains unclear and should be further investigated. Therefore, to determine whether surveillance CT has a role in detecting asymptomatic relapse and improving survival in patients with aNHL, we analyzed the data from our institutional lymphoma registry, which includes consecutively enrolled patients with lymphoma.

## Methods

### Patients

We retrospectively analyzed the data of patients who were enrolled consecutively in the Korea University Lymphoma Registry from June 1995 to October 2016. Three affiliated tertiary hospitals (the Anam, Guro, and Ansan hospitals) located in the metropolitan area participated in the registry. Patients who met the following inclusion criteria were selected: i) histologic diagnosis of aNHL (B-cell: diffuse large B-cell lymphoma [DLBCL], Burkitt lymphoma [BL], or B-cell lymphoblastic lymphoma; T-cell: peripheral T-cell lymphoma not otherwise specified [PTCL-NOS], angioimmunoblastic T-cell lymphoma [AITL], anaplastic large cell lymphoma [ALCL], NK/T-cell lymphoma [NKT], or T-cell lymphoblastic lymphoma [T-LL]); ii) patients who achieved a CR after frontline or salvage chemotherapy with curative intent; and iii) time from diagnosis to the last follow-up longer than 12 months. Pathological review was performed and aNHL was diagnosed as defined according to 2008 World Health Organization criteria [14]. All patients were staged in accordance with the Ann Arbor staging system [15]. To assign a risk score for aNHL, we calculated the International Prognostic Index (IPI) score [16] and dichotomized the scores into low risk (IPI 0–2) and high risk (IPI 3–5). The end-of-treatment response was evaluated within 1 month after completion of the intended chemotherapy. All responses were reassessed and the definitions of CR, partial remission (PR), stable disease, and progressive disease followed the Lugano classification [17]. If positron emission tomography (PET) was not performed, only CT scans were used to assess the response evaluation. The study was approved by the institutional review board of Korea University Hospital. All data were fully anonymized.

### Surveillance

**Fig 1:**
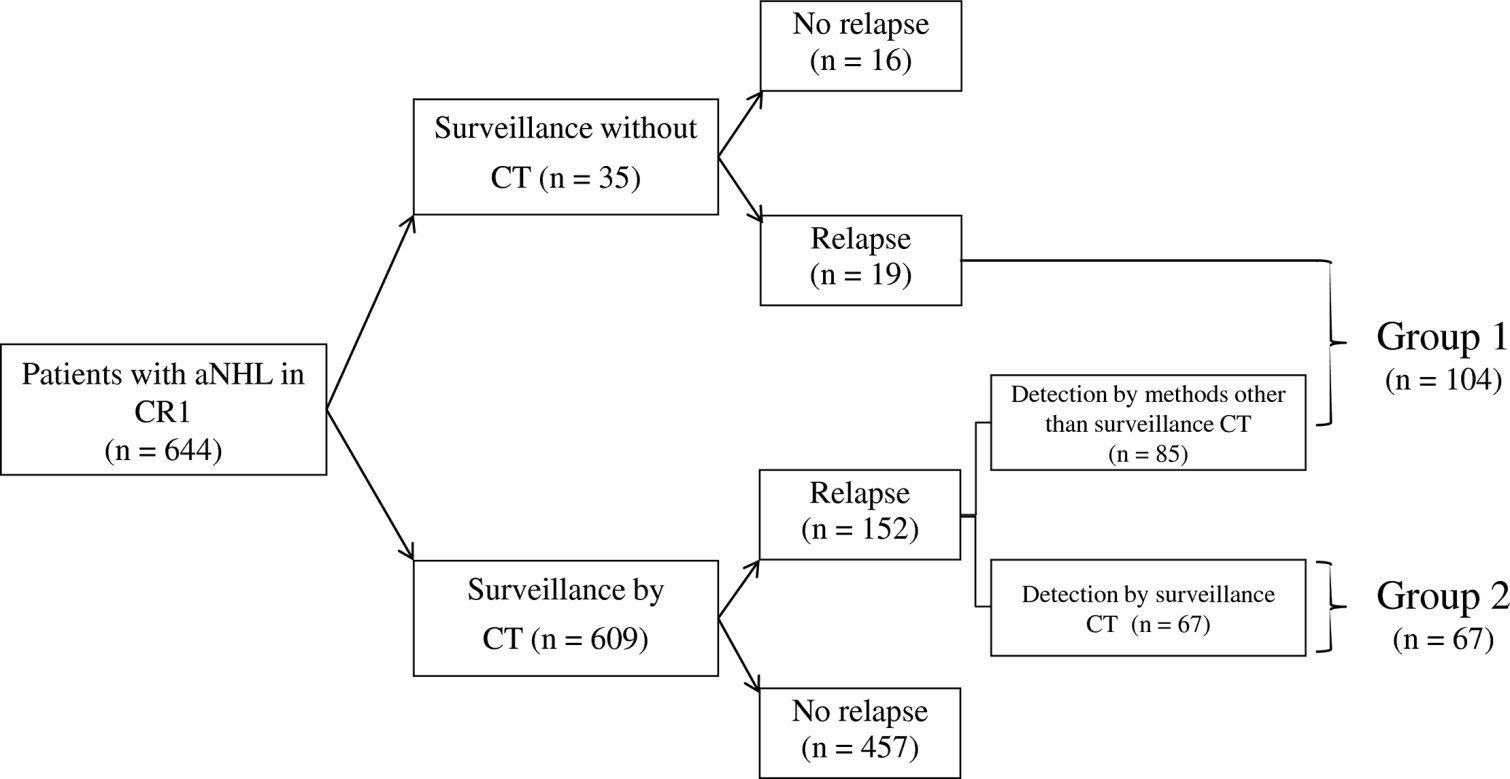
Diagram of the composition of patients in this study. Abbreviations: aNHL, aggressive non-Hodgkin lymphoma; CR1, complete remission after frontline chemotherapy; CT, computed tomography.

All patients in CR after frontline therapy were followed-up with clinical visits (symptom assessment, physical examination, and blood tests) every 1 to 6 months. Surveillance CT of the neck, chest, or abdomen was performed every 3 or 6 months or when clinically indicated for the first 2 years, and then every 6 or 12 months or when clinically indicated thereafter. Decisions regarding the surveillance strategy (clinical visit with appropriate blood chemistry, with or without regular surveillance CT) were at the discretion of the treating physician. Patients with identified relapse were categorized into two groups: group 1 consisted of patients in whom relapse was detected by methods other than surveillance CT (symptoms, physical examination, or blood tests), while group 2 consisted of asymptomatic patients in whom relapse was detected on surveillance CT (Fig 1). If clinical conditions were available, relapse was confirmed via biopsy of the suspected site. Although our institutions are divided into three independent tertiary centers, we use the same electronic chart program for drugs requiring caution. According to our regulations, all allergic reactions are to be recorded in the same format in the electronic chart program. Within this program, we also reviewed all medical records at the time of performing each CT scan. Adverse events related to the use of contrast media, such as contrast-induced nephropathy (CIN) and allergic-like reactions, were defined as below. CIN was defined as an increase from the baseline serum creatinine concentration of at least 0.5 mg/dL or at least 25% within 48 to 72 hours after exposure to contrast media and an allergic-like reaction was defined as acute if the anaphylactoid reactions, including rash, angioedema, bronchospasm, and cardiovascular collapse, or anaphylaxis occurred within 1 hour of contrast media administration.

### Statistical analysis

Mean values and standard deviations are reported for continuous variables and percentages for categorical values. Baseline characteristics at diagnosis and characteristics at relapse were analyzed by the Mann–Whitney and chi-squared tests, according to the type of variable. Overall survival (OS) was defined as the time period from diagnosis to death from any cause or censoring. Post-relapse survival (PRS) was defined as the time period from the date of identified relapse to death from any cause or censoring. OS and PRS were calculated using the Kaplan–Meier method and compared using the log-rank test. Multivariate and subgroup analyses for PRS were performed using the Cox proportional hazards method with the following variables from baseline: age, lymphoma subtype (DLBCL vs. PTCL), stage (limited vs. advanced), IPI risk group (low vs. high), bone marrow involvement, extranodal involvement, serum lactate dehydrogenase (LDH) level, interim response analysis during frontline therapy (CR vs. PR), autologous stem cell transplantation, and relapse detection method (group 1 vs. group 2). IBM SPSS version 21.0 (IBM Corp., Armonk, NY, USA) was used to analyze the data. A p-value of less than 0.05 was considered significant.

## Results

### Patient characteristics

A total of 1,385 patients with aNHL were included in the analysis. Patients with aNHL in the registry included 990 patients with B-cell aNHL (DLBCL [*n* = 960] and BL [*n* = 30]), and 395 patients with T-cell aNHL (PTCL-NOS [*n* = 96], AITL [*n* = 74], ALCL [*n* = 40], NKT [*n* = 157], and T-LL [*n* = 28]). Of these patients, 367 patients who had insufficient data (*n* = 142), received conservative treatment only (*n* = 143), were currently on frontline chemotherapy or the follow-up period from diagnosis was less than 12 months were excluded. Out of the 1018 patients who were able to be analyzed, 644 patients achieved CR after frontline chemotherapy (CR1). Those with B-cell aNHL included patients with DLBCL (*n* = 496/724 patients, CR1 rate: 68.5%) and BL (*n* = 11/16 patients, CR1 rate: 68.8%). T-cell aNHL included patients with PTCL-NOS (*n* = 22/59 patients, CR1 rate: 37.3%), AITL (*n* = 33/52 patients, CR1 rate: 63.5%), ALCL (*n* = 13/27 patients, CR1 rate: 48.1%), NKT (*n* = 63/120 patients, CR1 rate: 52.5%), and T-LL (*n* = 6/20 patients, CR1 rate: 30.0%). The median follow-up duration of patients in CR1 was 54.6 months (range: 12.2–226.0 months). In this cohort, 97.8% of B-cell aNHL was DLBCL and 95.6% of T-cell aNHL was PTCL (including PTCL-NOS, AITL, ALCL, and NKT). The baseline characteristics at diagnosis of patients who achieved CR1 are summarized in Table 1. Planned surveillance CT was performed in the majority of patients in CR1 (*n* = 609; 94.6%). In this study, the 5-year OS and 5-year relapse-free survival (RFS) of patients with DLBCL were 84.1% and 75.6%, respectively. The 5-year OS and 5-year RFS of patients with PTCL were 73.3% and 49.7%, respectively.

**Table 1:** Baseline characteristics at diagnosis in patients who achieved complete remission after frontline chemotherapy.

Baseline characteristics	All (*n* = 644)	DLBCL (*n* = 496)	PTCL (*n* = 131)*	*P*-value
Median age, years (range)	55 (14–85)	56 (14–85)	50 (17–85)	**< 0.001**^a^
Sex ratio (male:female)	1.40	1.37	1.43	0.850^b^
Disease stage, *n* (%)				0.396^b^
Limited (stages I–II)	426 (66.1)	325 (65.5)	91 (69.5)	
Advanced (stages III–IV)	218 (33.9)	171 (34.5)	40 (30.5)	
Extranodal involvement, *n* (%)	386 (59.9)	302 (60.9)	74 (56.5)	0.361^b^
Bone marrow involvement, *n* (%)	51 (8.2)	43 (8.9)	7 (5.5)	0.211^b^
B symptoms at baseline, *n* (%)	72 (11.2)	52 (10.5)	18 (13.7)	0.292^b^
LDH above normal limit, *n* (%)	243 (38.6)	200 (41.1)	36 (28.6)	**0.010**^b^
ECOG performance score, *n* (%)				0.192^b^
0–1	623 (96.7)	477 (96.2)	129 (98.5)	
2–4	21 (3.3)	19 (3.8)	2 (1.5)	
IPI score, *n* (%)				**0.001**^b^
< 3	524 (81.4)	389 (78.4)	120 (91.6)	
≥ 3	120 (18.6)	107 (21.6)	11 (8.4)	
HDT/ASCR, *n* (%)	51 (8.1)	31 (6.2)	17 (13)	**0.010**^b^
Surveillance CT, *n* (%)	609 (94.6)	470 (94.8)	122 (93.1)	0.627^b^

*PTCL includes peripheral T-cell lymphoma not otherwise specified, angioimmunoblastic T-cell lymphoma, anaplastic large cell lymphoma, and NK/T cell lymphoma.

Note: Bold indicates statistical significance.

^a^Mann–Whitney U test.

^b^Chi-square test.

Abbreviations: DLBCL, diffuse large B-cell lymphoma; PTCL, peripheral T-cell lymphoma; LDH, lactate dehydrogenase; ECOG, Eastern Cooperative Oncology Group; IPI, International Prognostic Index; HDT/ASCR, high-dose chemotherapy and autologous stem cell rescue; CT, computed tomography.

### Characteristics at relapse

In total, 644 patients achieved CR1 and 171 patients in this cohort relapsed (relapse rate: 26.5%) (Fig 1). The relapsed B-cell aNHL included DLBCL (*n* = 109/496 patients; 22.0%) and BL (*n* = 1/11 patients; 9.1%). The relapsed T-cell aNHL included PTCL-NOS (*n* = 10/22 patients; 45.5%), AITL (*n* = 13/33 patients; 39.4%), ALCL (*n* = 5/13 patients; 38.5%), NKT (*n* = 29/63 patients; 46.0%), and T-LL (*n* = 4/6 patients; 66.7%). A summarized diagram of the composition of patients in CR1 is presented in Fig 1. Of the 609 patients in whom surveillance CT was performed, 152 relapsed. Asymptomatic relapse was detected using surveillance CT in 67 patients (44%) and relapse outside the interval of surveillance CT was identified in the other 85 patients (56%).

Thus, of the 171 cases of relapse that were identified among patients with aNHL in CR1, 104 (104/171; 61%) were detected using methods other than surveillance CT. The chief complaints of patients at relapse included lymphadenopathy (35/104 patients; 33.7%), pain (33/104 patients; 31.7%), central nervous system symptoms (12/104 patients; 11.5%), B symptoms (11/104 patients; 10.6%), skin nodules (7/104 patients; 6.7%), and other symptoms (ulcer, edema, bleeding, etc.; 6/104 patients; 5.8%). Abnormally high serum LDH levels were detected in 76 patients (45.8%) (Table 2). In group 2, a median of three follow-up CT scans (range: 1–15 CT scans) were performed in order to detect a single relapse.

**Table 2:** Characteristics at relapse according to method of detection in relapsed aNHL patients after CR1.

Characteristics at relapse	All (*n* = 171)	Group 1* (*n* = 104)	Group 2* (*n* = 67)	*P*-value
Biopsy-proven relapse, *n* (%)	109 (63.7)	72 (69.2)	37 (55.2)	0.063^a^
Median time to relapse, months (range)	10.0 (1.2–97.4)	10.1 (1.2–97.4)	9.9 (2.2–49.5)	0.399^b^
Visit or surveillance interval, *n* (%)				**< 0.001**^a^
< 3 months	74 (43.3)	74 (71.2)	0 (0.0)	
Every 3 months	75 (43.9)	27 (26.0)	48 (71.6)	
Every 6 months	22 (12.9)	3 (2.9)	19 (28.4)	
Time to relapse, *n* (%)				
1st year of follow-up	91 (53.2)	54 (51.9)	37 (55.2)	0.673^a^
2nd year of follow-up	38 (22.2)	16 (15.4)	22 (32.8)	**0.007**^a^
3rd year of follow-up	17 (9.9)	14 (13.5)	3 (4.5)	0.055^a^
After 3rd year of follow-up	25 (14.6)	20 (19.2)	5 (7.5)	**0.033**^a^
Relapse site, *n* (%)				**< 0.001**^a^
Lymphoma-involved fields at diagnosis	83 (48.5)	39 (37.5)	44 (65.7)	
Other fields	88 (51.5)	65 (62.5)	23 (34.3)	
Histology, *n* (%)				0.107^a^
DLBCL	109 (63.7)	60 (57.7)	49 (73.1)	
PTCL^†^	57 (33.3)	41 (39.4)	16 (23.9)	
Other	5 (3.0)	3 (2.9)	2 (3.0)	
Extranodal involvement, *n* (%)	104 (60.8)	76 (73.1)	28 (41.8)	**< 0.001**^a^
Bone marrow involvement, *n* (%)	9 (9.6)	8 (13.6)	1 (2.9)	0.088^a^
LDH above normal limit, *n* (%)	76 (45.8)	46 (45.5)	30 (46.2)	0.939^a^
ECOG performance score, *n* (%)				0.115^a^
0–1	135 (78.9)	78 (75.0)	57 (85.1)	
2–4	36 (21.1)	26 (25.0)	10 (14.9)	
Disease stage, *n* (%)				0.980^a^
Limited (stages I–II)	107 (62.6)	65 (62.5)	42 (62.7)	
Advanced (stages III–IV)	64 (37.4)	39 (37.5)	25 (37.3)	

*Patients with relapse detected by methods other than surveillance CT comprise group 1; patients with relapse detected by surveillance CT scans comprise group 2.

†PTCL includes peripheral T-cell lymphoma not otherwise specified, angioimmunoblastic T-cell lymphoma, anaplastic large cell lymphoma, and NK/T cell lymphoma.

Note: Bold indicates statistical significance.

^a^Chi-square test.

^b^Mann–Whitney U test.

Abbreviations: CR1, complete remission after frontline chemotherapy; DLBCL, diffuse large B-cell lymphoma; PTCL, peripheral T-cell lymphoma; LDH, lactate dehydrogenase; ECOG, Eastern Cooperative Oncology Group; CT, computed tomography.

Patient characteristics at relapse are summarized in Table 2. There was no significant difference between the groups in median time to relapse from the end-of-treatment response (10.1 months vs. 9.9 months for groups 1 and 2, respectively; p = 0.399). Most relapses (129/171 patients; 75.5%) occurred within 2 years after achieving CR with frontline therapy; however, 24.5% of relapse cases were detected during a longer follow-up period. In group 1, relapses were detected more frequently in fields other than the originally lymphoma-involved fields (62.5% vs. 37.5%). By contrast, in group 2, relapses were more frequently detected in the original lymphoma-involved fields than in other fields (65.7% vs. 34.3%; p < 0.001). At the time of relapse, group 2 showed significantly lower extranodal involvement (p < 0.001). In group 2, extranodal involvements were observed in the muscle/bone (12/28 patients; 42.9%), solid organs (8/28 patients; 28.6%), hollow viscus (6/28 patients; 21.4%), and breast (2/28 patients; 7.1%). In group 1, the proportion of patients with extranodal involvement in the muscle/bone (28/76 patients; 36.8%) was similar to that in group 2, while the proportions of extranodal involvement of the solid organs (9/76 patients; 11.8%), hollow viscus (8/76 patients; 10.5%), and breast (3/76 patients; 3.9%) were lower than those in group 2. Instead, there were more patients with extranodal involvements of the brain (15/76 patients; 19.7%), skin (10/76 patients; 13.2%), and oral mucosa (3/76 patients; 3.9%). No significant differences were found between groups among other characteristics at relapse, including tumor histology, bone marrow involvement, LDH level, Eastern Cooperative Oncology Group performance score, and disease stage.

### Survival analysis: Overall survival and post-relapse survival

**Fig 2:**
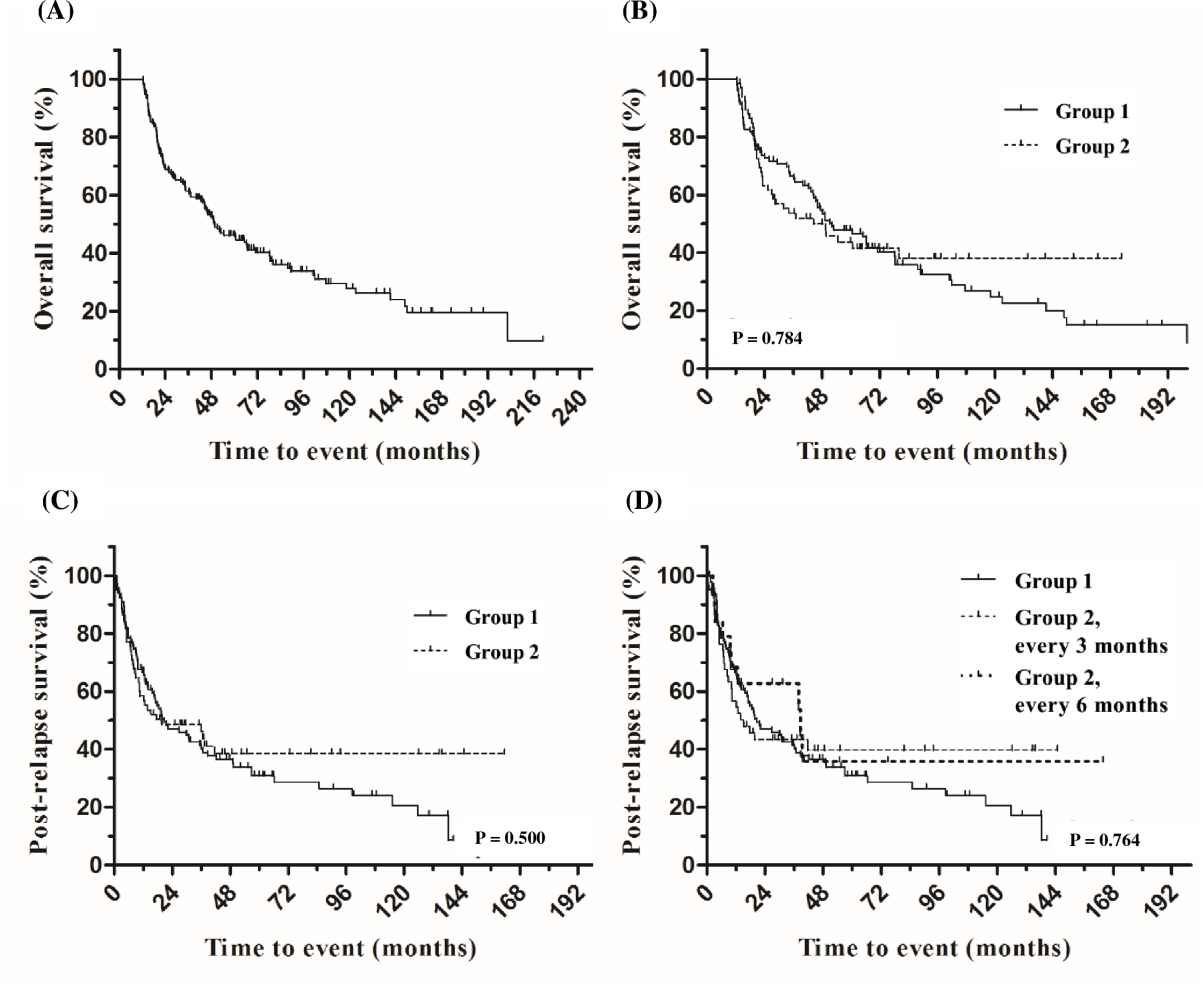
Kaplan-Meier curves of overall survival and post-relapse survival in relapsed aNHL patients after achieving complete remission with frontline chemotherapy. (A) Overall survival in 171 patients who relapsed after achieving complete remission with frontline therapy. (B) Overall survival according to relapse detection method. (C) Post-relapse survival according to relapse detection method. (D) Post-relapse survival according to interval of surveillance computed tomography (CT) scan. Group 1: Relapse detected by a method other than CT (symptoms, physical examination, or blood tests). Group 2: Relapse detected by surveillance CT.

The median OS of patients with aNHL in CR1 was 202 months (95% confidence interval [CI]: 117.6 to 286.8 months). The median OS of patients with aNHL with relapse was 49.3 months (95% CI: 37.6 to 61.1 months) (Fig 2A). There was no significant difference in the OS of relapsed aNHL patients after CR1 according to the relapse detection method (group 1 vs. group 2; p = 0.784) (Fig 2B).

No significant difference was observed in PRS between groups (20.6 months, [range: 9.3–31.9 months] in group 1 vs. 19.6 months [range: 0.0–45.0 months] in group 2; p = 0.500) (Fig 2C). Moreover, there was no significant difference in the median PRS according to the interval (3 or 6 months) of surveillance CT (p = 0.764) (Fig 2D). In multivariate analysis for PRS, age (≥ 60 years) and advanced disease stage at diagnosis (stages III or IV) were independently associated with poor PRS (hazard ratio [HR] 1.81, 95% CI: 1.18 to 2.80; p = 0.007 and HR 1.62, 95% CI: 1.08 to 2.43; p = 0.020, respectively). The surveillance strategy (group 1 vs. group 2) did not affect PRS in multivariate analysis.

**Fig 3:**
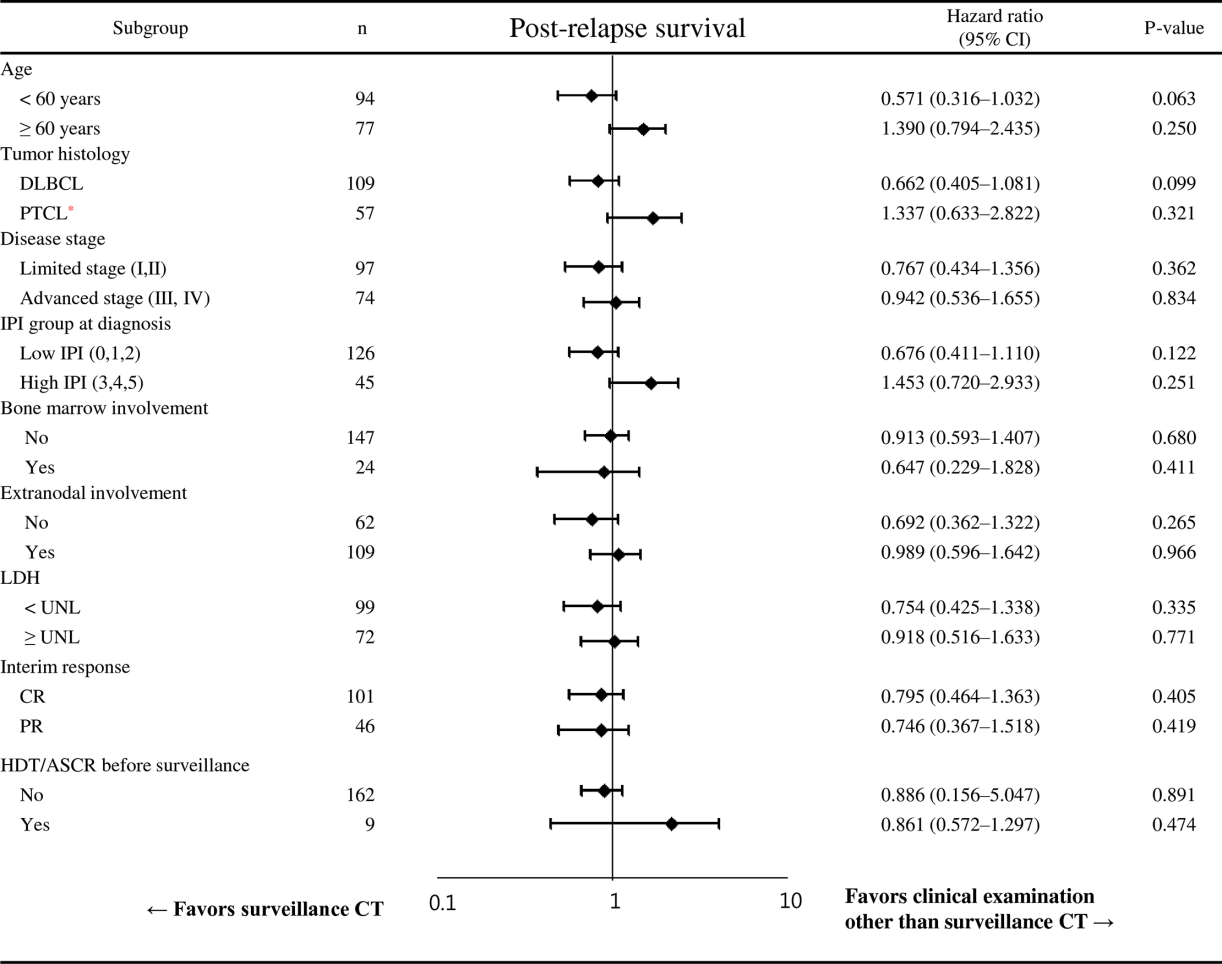
Subgroup analysis of post-relapse survival according to relapse detection method in relapsed aNHL patients after CR1. *PTCL includes peripheral T-cell lymphoma not otherwise specified, angioimmunoblastic T-cell lymphoma, anaplastic large cell lymphoma, and NK/T cell lymphoma. Abbreviations: CR1, complete remission after frontline chemotherapy; CI, confidence interval; DLBCL, diffuse large B-cell lymphoma; PTCL, peripheral T-cell lymphoma; LDH, lactate dehydrogenase; IPI, International Prognostic Index; UNL, upper normal limit; CR, complete remission; PR, partial remission; HDT/ASCR, high-dose therapy with autologous stem cell rescue.

To investigate the impact of the frequency of clinical visits (visit interval < 3 months), we analyzed PRS among patients with clinical visit or surveillance intervals ≥ 3 months (i.e., 3 or 6 months). There was no significant difference between groups (group 1 [n = 30] 30.8 months, 95% CI: 6.6–54.9 months vs. group 2 [n = 67] 19.6 months, 95% CI: 0–45 months; p = 0.404]. In subgroup analysis including age, tumor histology, stage, IPI group at diagnosis, bone marrow involvement, serum LDH level, interim response during frontline therapy, and previous autologous stem cell transplantation, no subgroup favored the use of surveillance CT among relapsed aNHL patients after CR1 (Fig 3).

**Fig 4:**
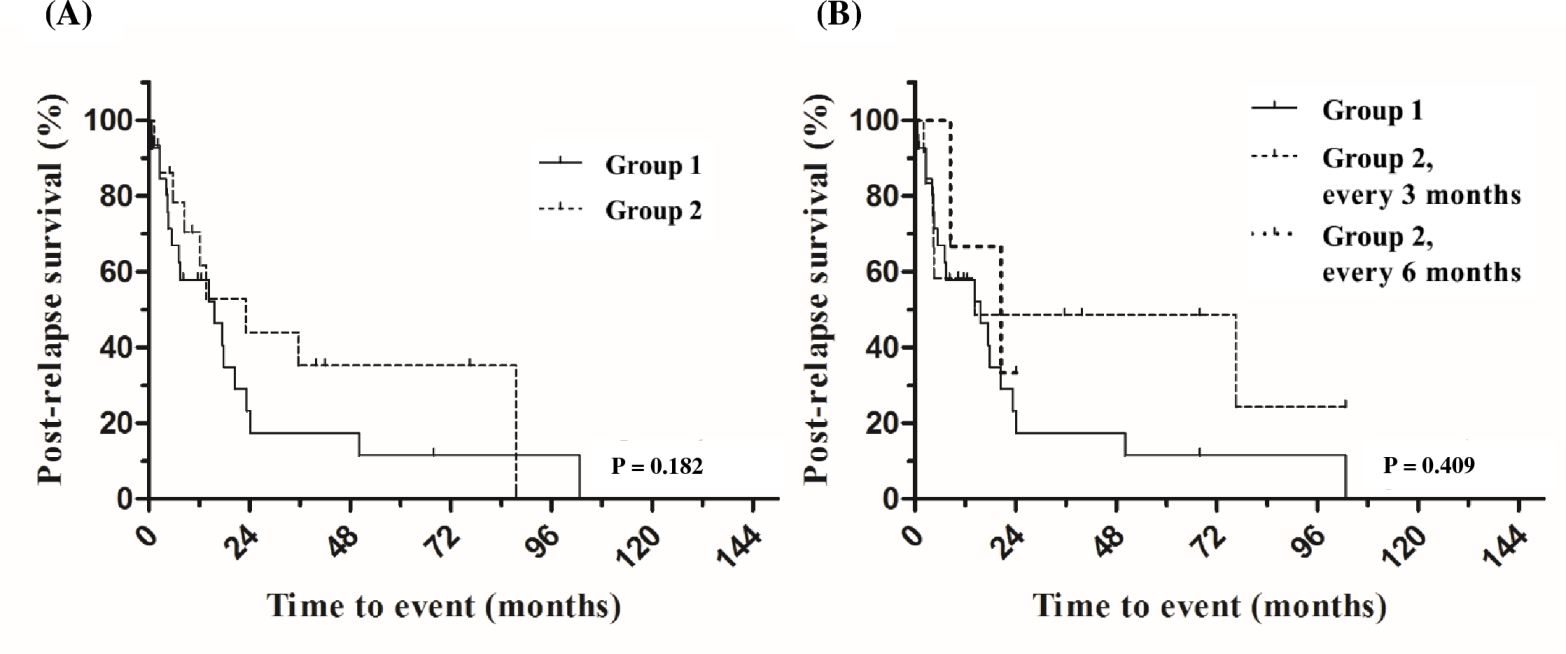
Kaplan-Meier curves of post-relapse survival in relapsed aNHL patients after achieving complete remission with salvage chemotherapy. Post-relapse survival according to (A) relapse detection method and (B) surveillance CT interval among patients who relapsed after achieving complete remission with salvage therapy. Group 1: Relapse detected by a method other than CT (symptoms, physical examination, or blood tests). Group 2: Relapse detected by surveillance CT.

Additionally, we analyzed the impact of surveillance CT in patients with refractory or relapsed aNHL who achieved CR after salvage chemotherapy (CR2). Of 315 patients with aNHL that had relapsed or were refractory to frontline chemotherapy (171 relapsed and 144 refractory patients), 99 achieved CR after salvage chemotherapy (81 relapsed and 18 refractory patients) and were followed with clinical visits with or without surveillance CT. Relapse was detected in 42 patients (42/99; 42.4%). A total of 27 (64.3%) and 15 patients (35.7%) were identified as relapsed by methods other than CT scan and using surveillance CT, respectively. There was no significant difference in the median PRS between the two groups (12.5 months, 95% CI: 2.8 to 22.1 months vs. 10.7 months, 95% CI: 0 to 41.5 months; p = 0.182] (Fig 4A). Similar to our observations in patients in CR1, the surveillance CT interval (3 or 6 months) did not affect the PRS of patients with aNHL in CR2 (p = 0.409) (Fig 4B).

### Adverse events related to the use of contrast media

In patients who underwent regular surveillance CT scans after achieving CR1 (*n* = 609), 28 experienced more than one CIN (28/609; 4.60%) and 15 complained more than once of allergic-like reactions (15/609; 2.46%). Each CIN and allergic-like reaction required short-term interventions (hydration, anti-histamine agents, and/or steroids) but there were no severe adverse events that required long-term interventions, including CIN requiring dialysis or anaphylactic shock. CIN was not correlated with relapse (CIN -: 26.5% vs. CIN +: 28.6%, p = 0.805) and allergic-like reactions did not affect the relapse rate (allergic-like reactions -: 26.6% vs. allergic-like reactions +: 28.7%, p = 0.992). CIN or allergic-like reaction did not affect OS (p = 0.600, and p = 0.854, respectively).

## Discussion

In this study, of the 644 patients with aNHL in CR1, relapse was detected in 171 (overall relapse rate, 26.6%). Asymptomatic relapse was detected in less than half of the total relapsed patients (n = 67; 44%), although the planned surveillance CT was performed in the majority of patients (n = 609; 94.6%). The surveillance strategy did not affect OS or PRS in patients with relapsed aNHL after CR1 and CR2. No subgroups favored the use of surveillance CT among patients in CR1. Therefore, we did not observe a benefit for detecting asymptomatic relapse using surveillance CT in this study. Moreover, the interval of clinic visits or CT did not affect PRS.

Currently, the National Comprehensive Cancer Network guideline for DLBCL recommends surveillance CT no more than every 6 months for 2 years after completing therapy in patients with stage III–IV DLBCL [3]. European guidelines for DLBCL and PTCL established by the European Society for Medical Oncology only state that “minimal surveillance CT scans at 6, 12, and 24 months after end of treatment constitutes usual practice;” the guidelines give no recommendation [1, 2]. However, our results suggest that surveillance CT has no survival benefit for patients with aNHL after curative chemotherapy, irrespective of stage, tumor histology, and CT interval. This finding is consistent with the American Society of Hematology Choosing Wisely campaign to “limit the use of surveillance CT in asymptomatic patients with aNHL after curative-intent treatment” [18].

Previously, several studies examined the role of surveillance imaging in patients with lymphoma [6, 7, 19–24]. However, in most of these studies, the study population included patients with indolent lymphoma as well as aNHL and PET or combined PET/CT was included as surveillance imaging. Currently, it is generally accepted that the role of surveillance PET/CT is limited among patients with aNHL in CR1 owing to a high rate of false positivity [25–27]. Therefore, this study was designed to include only aNHL patients who underwent curative chemotherapy in whom only CT was performed as imaging during surveillance. The use of surveillance CT in detecting asymptomatic relapse was shown to be ineffective in this study. Asymptomatic relapse was detected in less than half of the total relapsed patients (67/171; 44%). This represents 11% of all patients who underwent surveillance CT. This finding is consistent with the results of previous studies; detection rates of 5.7% to 38% have been reported for asymptomatic relapse using surveillance CT in patients with aNHL [7, 19–24].

Most previous studies on surveillance CT among patients with aNHL in CR revealed that the detection of asymptomatic relapse in these patients did not contribute to improved survival [6, 20, 21, 23]. These results are consistent with those of the present study, which showed that surveillance strategy did not affect OS and PRS in this group of patients. Similarly, in a large Danish-Swedish population-based cohort study (525 Danish patients who underwent surveillance CT and 696 Swedish patients who did not undergo surveillance CT), there was no significant difference in post-treatment survival between the Danish and Swedish cohorts. These findings were also reported in a subgroup of patients in the IPI risk group [6]. Similarly, we did not identify any subgroup of patients for which surveillance CT was favorable for PRS. One study showed that imaging-detected relapse was associated with a lower disease burden and a possible survival advantage [22]. However, we observed no significant difference between groups by stage at relapse (Table 2). In a study by El-Galaly et al. in which a considerable portion (17%) of the study population included patients with Hodgkin lymphoma, it was shown that, after excluding patients with indolent histology, the survival benefit was not maintained [22]. This finding is consistent with the results of most studies that have investigated the role of surveillance CT in survival, including ours. It can therefore be considered that no current studies suggest a survival advantage through the use of surveillance CT in patients with aNHL who have achieved CR after curative chemotherapy. Moreover, we also observed similar results in relapsed/refractory patients who had been cured after salvage chemotherapy. These findings suggest that the window of opportunity for detecting asymptomatic relapse is smaller than expected. This is supported by the finding of this study that a more frequent surveillance CT scan interval did not improve PRS (Figs 2D and 4B). Actually, aNHL is often accompanied by rapidly developing symptoms. The present study results suggest that a longer follow-up period of more than 2 years should be required, because approximately one-third of patients experienced relapse 2 years or more after the completion of chemotherapy.

In this study, asymptomatic patients in whom relapse was detected on surveillance CT scans showed lower extranodal involvement. There are several possible explanations for this observation. First, it is possible that the scope of the CT scans was not sufficient to detect whole extranodal relapse. In our study, about 15% of patients who relapsed after achieving CR1 (25/171 patients) had extranodal relapse detected by symptoms, including central nervous system symptoms, skin nodules, edema of the extremities, and ulcer or bleeding of the gastrointestinal tract. CT scans covering the neck, chest, and abdomen have limitations for the identification of relapse in these sites, and this could be the reason that the surveillance CT scan group showed lower extranodal involvement. Second, different pathophysiology might exist between the relapses detected by clinical manifestation and surveillance CT. Aggressive lymphomas can be accompanied by rapidly growing masses and fulminant symptoms, often with extranodal involvement [28]. It is likely that more aggressive lymphomas are detected clinically, which may explain why patients in whom relapse was detected by methods other than surveillance CT (symptoms, physical examination, or blood tests) had higher extranodal involvement.

The causal relationship between radiation and cancer has been established from data obtained from atomic bomb survivors [29]. Because the radiation dose from repeated surveillance CT is usually in the low-dose range (less than 1000 mSv), it is important to investigate whether this relationship remains pertinent in patients exposed to low-dose radiation. One study showed a significantly increased risk of cancer in the range of 0–1000 mSv, with a potential calculated threshold was 600 mSv [30]. Accordingly, it has been estimated that approximately 0.4% of cancers in the United States and 0.6% of cancers in the United Kingdom may be attributable to radiation from CT [31, 32]. Therefore, the risk of cancer associated with CT is not hypothetical and exposure to low-dose radiation from CT should be minimized as much as possible.

The adverse effects of contrast media, such as CIN and allergic-like reactions, should also be considered. With low- or iso-osmolar contrast media, the incidence of CIN and allergic-like reactions has been shown to be 0.7–3.1% [33] and 1.2–1.6% [34] in the general population, respectively. In this study, the allergic-like reaction rate (2.46%) was within the reported range; however, the incidence of CIN (4.60%) was higher than that in the general population. In a study by Launay-Vacher et al., renal insufficiency was common in patients with cancer [35]; this may have been because advanced age, comorbidities, and use of concurrent nephrotoxic drugs are common in these patients. As pre-existing renal insufficiency is the most important risk factor for CIN [36], patients who have been treated with nephrotoxic chemotherapy agents could have a higher risk for CIN.

The strengths of this study include the population consisting of patients with aNHL without indolent histology and that the surveillance imaging was limited to CT without including PET/CT. Moreover, the timing of surveillance CT was relatively controlled over the surveillance period (3- or 6-month intervals). The relapse characteristics were summarized from the registry, which consisted of consecutive patients with lymphoma over a 12-year period. However, this study has some limitations. First, the number of pathologic confirmations at the time of relapse was relatively small (n = 109; 63.7%), although relapses were comprehensively assessed by clinical symptoms, blood tests, and imaging in the other 62 patients. Thus, it is possible that patients with false positive results might have been included. Above all, the findings of this study should be interpreted carefully because this is a retrospective analysis.

In conclusion, the results of this study suggest that routine surveillance CT among patients with aNHL in CR might have a limited role in improving survival. The beneficial effects of surveillance imaging have not been reported in most studies, including the present study. There are potential risks of CT due to exposure to ionizing radiation and contrast media, among others. It is, therefore, desirable to avoid repetitive CT for detecting asymptomatic relapse among aNHL patients in CR until the advantage of surveillance CT is supported by a high level of evidence. CT to identify relapse would only be appropriate when relapse is clinically suspected.
